# Explain the challenges of evacuation in floods based on the views of citizens and executive managers

**DOI:** 10.1016/j.heliyon.2022.e10759

**Published:** 2022-09-25

**Authors:** Changiz Ahmadi, Arezou Karampourian, Mohammad Reza Samarghandi

**Affiliations:** a Hamadan University of Medical Sciences, Hamadan, Iran; b Urology and Nephrology Research Center, Chronic Diseases (Home Care) Research Center, School of Nursing and Midwifery, Hamadan University of Medical Sciences, Hamadan, Iran; c Department of Environmental Health, School of Health, Health Sciences Research Center, Health Sciences & Technology Research Institute, Hamadan University of Medical Sciences, Hamadan, Iran

**Keywords:** Natural disasters, Floods, Disaster medicine, Qualitative research

## Abstract

**Background:**

Flood is one of the natural hazards that causes a lot of human and financial losses. Emergency evacuation in the response phase is necessary to reduce damage. The present study was conducted to explain the challenges related to evacuation in floods based on the views of citizens and executive managers.

**Materials and methods:**

In this study, a qualitative research method with a contractual content analysis approach was used. 27 participants including 10 citizens and 17 executive managers were included in the study by purposive sampling. Semi-structured interviews were used to collect data. In order to have confidence the validity of the results, rigor was certified by using the guidelines suggested by Guba and Lincoln for making trustworthiness. The duration of the interview was between 45 min and 60 min. The interviews were handwritten by line-by-line content analysis and then analyzed.

**Results:**

Data analysis led to the extraction of 6 main categories with 14 sub-categories including: lack of primary warning system (with 2 sub-categories, lack of national early warning system and lack of attention to early warning), insufficient resources (with 4 sub-categories, lack of equipment, lack of manpower Human resources, inadequate allocation of financial resources and lack of information resources), problems related to unpreparedness (having 2 sub-categories, lack of pre-determined program and lack of attention to public education), problems related to emergency housing (having 2 sub-categories, lack of housing program and lack of attention to indigenous culture in housing), lack of risk perception (has 2 sub-categories of people’s belief in floods and officials' belief in flood) and problems related to lack of coordination (has 2 sub-categories of internal disharmony and external disharmony).

**Conclusion:**

To increase evacuation, it is necessary to identify the relevant challenges. Establishing an early warning system and evacuation plan, supply of resources, and increase risk perception and coordination can increase the speed of evacuation and reduce the financial and human losses caused by floods.

## 1 Introduction

The floods are the most widespread natural hazard in the universe [1]. Asian countries are more affected by the effects of floods [2, 3]. More than half of the world’s floods occur in Asia. Iran is one of the countries exposed to floods. The great flood of 2019 in Iran will affect 25 of the 32 provinces [1]. Changes in climate and rainfall have increased the number of floods and the severity of their effects in the world [2]. The flood has numerous human, economic, social, cultural and social consequences [3]. The floods are the most expensive and devastating natural disasters have short-term and long-term effects [4] that impress many person each year [5]. The worst consequences of floods are death and the spread of infectious diseases. About 196 million people in more than 90 countries are at risk each year [3]. Floods cause significant economic damage by destroying agricultural land and destroying livestock. Floods can also cause people to migrate due to business losses and create marginalization. Marginalization will create cultural confrontation [6]. Flood damage was estimated at $ 14 billion between 1980 and 1990, while it reached $ 100 billion between 2004 and 201 [3].

One of the aims of The Sendai Framework for Disaster Risk Reduction 2015–2030 is to reduce the risk of natural hazards and its effects on human life [5]. This international document (the Sendai Framework) is the first main agreement on the post-2015 development agenda and prepares member states with concrete measures to safeguard development gains against disaster risk [7].

Risk management policies and interventions reduce flood mortality [8]. In order to prevent the reduction of the probability of flood consequences, in addition to structural measures such as the construction of a dam, non-structural measures such as forecasting, warning and evacuation can be used [9]. The response phase, as one of the phases of disaster management, includes measures to manage and control the various effects of disasters and minimize human and property losses. These measures include flood forecasting, flood control operations and evacuation [10]. Evacuation is a response that must be done quickly and effectively and is essential to relocating residents from high-risk areas to safer places. Emergency evacuation is essential to protect the health and lives of people, animals, historical sites and documents. There are two types of evacuation orders: mandatory and voluntary. In emergencies, people are reluctant to evacuate because their property is not protected, so they prefer to decide to evacuate themselves [11].

Various quantitative studies have been carried out in the field of flood management such as flood risk assessment and forecasting [12, 13, 14]. However, fewer studies have comprehensively and qualitatively addressed flood response especially evacuation. Also previous studies have investigated the influence of human behaviors on evacuation processes, however studies that analyze the influencing factors are limited [15].

One of the effective methods and strategies to reduce flooding is awareness of quality and understanding of public risk [16]. Knowing the public perception of risks shows people’s desire to take preventive measures and society’s support for government risk reduction policies [17].

In the flood of Poldokhtar, one of the cities of Iran, it was observed that despite the evacuation order to the people along the roads, it was not evacuated. These is a scarcity of qualitative data regarding evacuation from different relevant perspectives. Therefore, investigating explain the challenges of evacuation in floods based on the views of citizens and executive managers help us to gain a deeper understanding of these challenges.

## 2 Materials and methods

### 2.1 Design

In this study, qualitative content analysis study with conventional approach was used. The study, qualitative content analysis, is an appropriate method for causing knowledge, new ideas, and practical guidance to get the goal of this research [18, 19].

The steps involved in conducting the study include: recording all interviews, transcription, coding. Then, data analysis was performed to extract and categorize the challenges of evacuation in floods based on the views of citizens and executive managers using conventional content analysis method in which coding categories are derived directly from the text data. Also Code counting and checking their frequency was done for challenges of evacuation in floods based on the views of citizens and executive managers. The following paragraphs illustrate how the research method works:

### 2.2 Data collection or data generation

Semi-structured interviews were used to collect data. Open-ended questions were used to collect experiences [20]. Interviews continued until information saturation [21]. The duration of the interview was between 45 min and 60 min based on the tolerance, amount of information, willingness and agreement of the participants. The interviews were conducted individually at a time and place acceptable to the participants. Interviews were conducted in the homes of citizens and executive managers' workplaces. Before starting each interview, participants were talked about the purpose and importance of this study. Interviews with participants were recorded and based on the main research question. Some questions include the following section:

Please share your experience of the flood. Have you ever experienced a flood? What problems did you have for emergency evacuation in the flood? What problems did the organizations have in evacuating the people? What are the factors affecting the emergency evacuation of people? What is your suggestion to managers and organizations for emergency evacuation? What do you suggest to people for emergency evacuation? The following exploratory questions were also used: “Please explain more? What did you mean? Why?”

Data collection continued until the data was repeated and no new code was extracted, this state is called data saturation [21]. Interviews were quickly implemented and typed using Word office software. It should be noted that to confirm the study, two persons coded all interviews, and eventually the codes and categories were reformed, combined or deleted by a disaster and public health specialist.

### 2.3 Data analysis

Data analysis was performed according to the data obtained from qualitative stage analysis. Categories and sub categories were extracted from the primary data by careful review and continuous comparison of data. The data analysis was done based on the content analysis principles proposed by Graneheim and Lundman [22, 23, 24]. In the first step, the interviews were frequently listened and transcribed word by word. In the second step, the texts were read many times to understand the ideas of the participants. In the third step, the interviews were considered as a whole, and the main gist of the context the fundamental meaning or was described as a whole in one or more segment. In the fourth stage, the themes, codes, and initial categories were designed. Then, after sequential analyses and systematic comparisons, categories, sub categories, themes, and repetitive codes were merged into each other for providing a proper classification of them.

### 2.4 Trustworthiness

In order to have confidence the validity of the results, rigor was certified by using the guidelines suggested by Guba and Lincoln for making trustworthiness [25]. In order to obtain the requirement for credibility, the authors involved with the whole process the research. In addition, the essential investigator always engaged with depth interviews of the participants. Ongoing and prolonged engagement was ensured during the data collection and analysis. Triangulation was employed to ensure the confirmability and credibility of the data. The data collection and data analysis process were checked by two investigators during the coding process. In addition, a check was carried out by an expert in the field of disaster to validate the findings. Also, the process of coding and developing themes, and its monitoring, was accomplished by an expert in qualitative research to ensure the credibility of the data. Member checks were lead to ensure understanding and interactions between the researchers and participants. To the eligibility of the research, the team members had adequate experience in the field of disaster.

In addition, to transformability the findings, to maximize changes in sampling and selection methods, participants were from different organizations such as the Red Cross, the governor’s office, the municipality, and the Water and Sewerage Organization, and differed in terms of work experience, education, and gender. Finally, the experiences of citizens and managers were considered in this research. It should be noted that the generalizability of the study can be extended to flood-prone areas such as Poldokhtar with caution. The lessons learned, results, and the methodology of this study may be useful for mountainous cities with monsoon rains such as Poldokhtar.

In order to be reproducible, we documented the exact processes used.

### 2.5 Study setting

**Figure 1:**
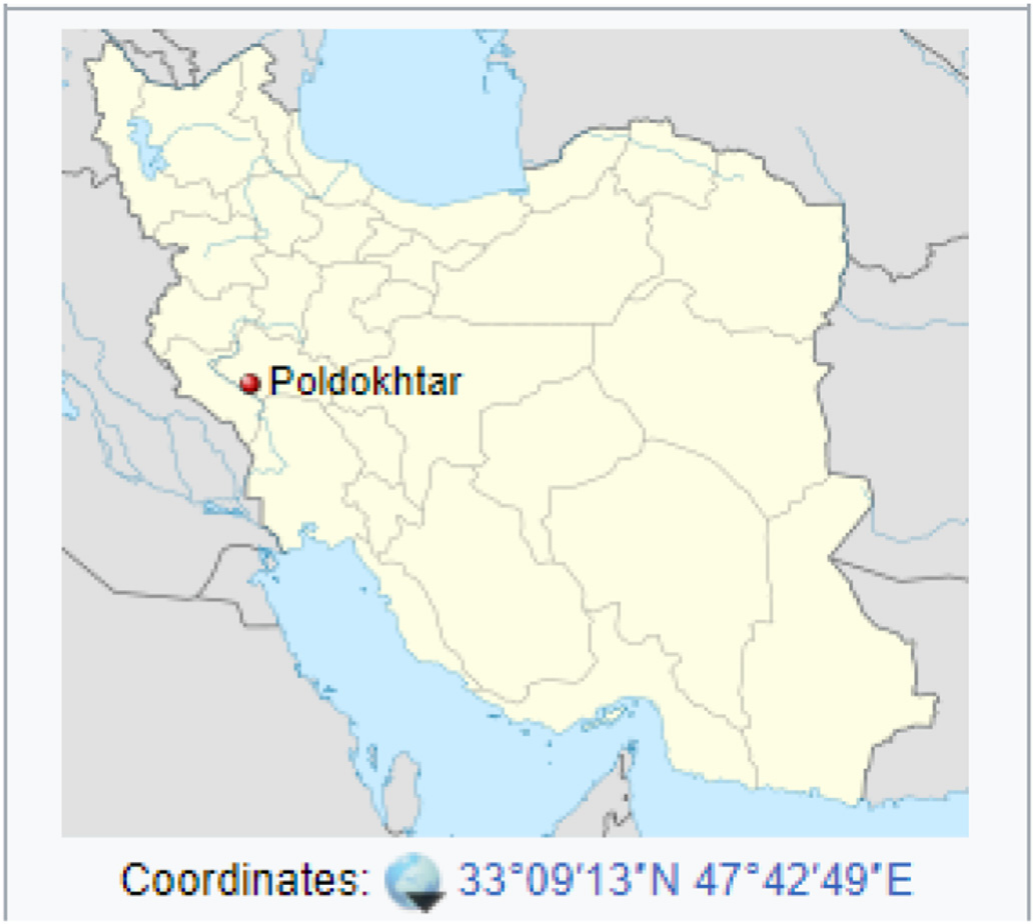
Map of Poldakhtar city.

This study was conducted in city of Poldokhtar in 2021. Poldokhtar is one of the cities of Lorestan province located in west of Iran (Figure 1).

The population of Poldokhtar city is 26,352 people. Keshkan River passes through the center of Poldokhtar city. In monsoons, this river overflows. Poldokhtar industries are mostly construction industries, the construction materials of which are also exported to other cities. Among these factories, sand, cement and plaster factories can be mentioned. The city was affected by awesome massive flood on April 2019.

### 2.6 Study participant

The study was conducted from the citizens of the coastal strip and the western side of Poldokhtar city who were exposed to floods due to geographical conditions, as well as executive managers who had experience in flood management. In this study, 27 participants including 10 citizens and 17 executive managers with flood experience were selected by purposeful sampling. Interviews were conducted in two groups: citizens and executive managers. Specifically, in selecting the citizens, the participants were selected from flood-affected areas among people. In selecting participants, it was tried to use the people involved in the flood. In selecting the executive managers, attempts were made to engage all who were someway involved in the management of disasters in the city. These people included those working in disaster management organizations such as the Red Crescent, the Governorate, the municipality, and the Water and Sewerage Organization. In selecting the participants, an attempt was made to observe maximum diversity in experiences. Inclusion criteria included having flood experience, ability to express and communicate, and willingness to participate in research.

### 2.7 Ethical considerations and study permission

This study was approved by the Ethics Committee under the code IRUMSHA.REC.1400.039 and number 140002281400. Written consent was obtained from participants to participate in the study. The interview time was arranged according to the coordination and request of the participants.

## 3 Results

The results showed that most of the participants were executive managers, male, average age of 39.11 ± 11.57, 82% had 5–10 years of disaster management experience (Table 1).

**Table 1:** Demographic characteristics of study participants.

	Variables	Number (Percent)
Participants	Executive managers Citizens	17 (62.96) 10 (37.04)
Age	<20 20–30 31–40 41–50 51–60	2 (7.41) 4 (14.81) 10 (37.04) 9 (33.33) 2 (7.41)
Sex	Male Female	25 (92.59) 2 (7.41)
Disaster management experience(year)	<5 10–5 11–15	2 (11.77) 14 (82.35) 1 (5.88)

The initial number of codes obtained from the interviews was 1251 codes (1183 related to managers and 168 related to citizens). The codes were first divided into 20 categories and 70 subcategories and after integration with inductive content analysis were divided into 6 main categories and 14 subcategories. The main theme of this study is coordination. This means that if there is coordination, along with the initial warning system, human/financial/information resources, emergency preparedness and resettlement program, there is an increase in risk perception among managers and citizens, flood management response will be possible. Also, the main categories of the creation of the early warning system (with 2 sub-categories, lack of national early warning system and ignoring early warning), inadequate resources (with 4 sub-categories, lack of equipment, lack of manpower, inadequate allocation of funds, and lack of information resources), problems related to unpreparedness (having 2 sub-categories, not having a pre-determined program and not paying attention to public education), problems related to emergency housing (having 2 sub-categories, lack of housing program and disregard for indigenous culture in housing), lack of perception of risk (with 2 sub-categories of people’s belief in floods and officials' belief in flood) and problems related to lack of coordination (with 2 sub-categories of internal disharmony and external disharmony) (Table 2).

**Table 2:** Explain the challenges related to flood evacuation.

Category	Category Subcategory	Codes
Ignorance of the creation of the early warning system	Lack of national early warning system	Inadequate early warning devices Lack of integrated early warning system Lack of national early warning code
	Ignoring the initial warning	People's distrust due to previous false warnings People ignore the initial flood warning
Inadequate resources	Lack of equipment	Shortage of equipment and heavy vehicles for dredging Shortage of trucks to move people/property/livestock Shortage of ambulance
	Lack of manpower	Shortage of expert rescuers Do not use native helpers familiar with the area
	Inadequate allocation of funds	Shortage of budget for the provision of evacuation equipment Lack of anticipation of accident insurance and tax forgiveness Lack of financial means to move
	Lack of information resources	Lack of meteorological technology Lack of alternative communication system Delay in communication system reconstruction
Problems of unpreparedness	Lack of evacuation schedule	Lack of flood response program/maneuvers Lack of emergency route forecasting Do not use the experiences of local trustees in planning
	Lack of attention to public education	Lack of public education Early warning Lack of public emergency evacuation training Lack of public first aid training
Emergency housing problems	Lack of accommodation schedule	Inadequate information about the availability of a safe place Lack of previous accommodation experience Ignore individual differences in the accommodation program
	Ignoring the indigenous culture in the settlement	People do not welcome temporary accommodation Ignoring the cultural characteristics of individuals Lack of trust in proper accommodation
Lack of risk perception	People believe in floods	Lack of trust in the media Not responding to the evacuation message
	Managers believe in floods	Inability to persuade people to evacuate Minimize the consequences of floods No evacuation of offices at risk
Problems of incoordination	Intra-organizational inconsistency	Lack of Incident Command System Do not use native/experienced managers
	Extra-organizational inconsistencies	Inconsistency in the division of tasks Occurrence of floods during the holidays Ignoring crises at the same time

### 3.1 Ignorance of the creation of the early warning system

The lack of an early warning system is one of the challenges of flood response. Data analysis revealed that participants indicated this challenge. Participants mentioned this many times.

#### 3.1.1 Lack of national early warning system

In data analysis, the majority of participants stated that one of the challenges of evacuation was the lack of a national and integrated early warning system. Dissemination of evacuation alerts in unknown ways such as ambulance sirens, fire engines, police, Red Crescent, each of which has a different meaning, is not only interpreted as an emergency evacuation warning, but also has different meanings. The shouts of rescue workers and the beating of houses cannot be the right way to respond. Given the history of the Iran-Iraq war and the well-known siren, the existence of a national system is necessary for the people’s trust.

“We warned with an ambulance siren, fire brigade and any device we had ... when we heard these sounds, we thought there was a fire somewhere or the ill patient was being dispatched or the accused was fleeing .....”

#### 3.1.2 Ignoring the initial warning

In data analysis, the majority of participants mentioned that one of the challenges of not evacuating was not trusting the initial warning. People have seen false warnings many times, and this has led to their distrust.

“Several times we were informed that a big flood was happening and we were preparing ourselves but we saw that it was not a flood and that made us distrust .....”

### 3.2 Inadequate resources

The lack of necessary resources, equipment and facilities for evacuation was one of the other challenges that the participants repeatedly mentioned.

#### 3.2.1 Lack of equipment

According to most of the participants, they mentioned the shortage and breakdown of heavy machinery. People did not have vehicles to move themselves and their livestock.

“We did not have a heavy machine for dredging ... if our property and livestock are to be destroyed, it is better that we are not alive ...”

#### 3.2.2 Lack of manpower

According to most of the participants, most of the people lacked skilled and local helpers and the helpers were not familiar with the area.

“We needed skilled people to move, but the rescuers did not know the area at all and were not natives ...”

#### 3.2.3 Inadequate allocation of funds

According to most participants, the lack of flood insurance and tax forgiveness, fear of thieves, financial poverty for transfer costs, and unfair distribution of resources were the evacuation challenges.

“We have no tax exemption or forgiveness and we are afraid that if we leave our house and shop, thieves will rob us ...”

#### 3.2.4 Lack of information resources

According to most participants, the lack of a national information channel delayed the publication of the news. Delay in warning also causes distrust among the people. Contradictory, scattered, and late news also causes people not to be persuaded to evacuate. Meanwhile, due to the disruption of the telecommunication system, the people were both unaware of the relatives' situation and could not receive their help.

“The telephones were cut off and we were unaware of the relatives and could not say we wanted help ...... there was no system to register missing people ... we did not receive the news correctly and on time, everyone was saying something. ....”

### 3.3 Problems of unpreparedness

Lack of preparation was one of the problems that citizens and executive managers were dealing with.

#### 3.3.1 Lack of evacuation schedule

According to most of the participants, Poldokhtar had a history of monsoon floods, but despite this, there was no plan to deal with the flood, monitor the construction of the river, predict the emergency route and pay attention to previous lessons learned. The experiences of trusted and experienced seniors and the elderly were not used in the decisions.

“We have elderly people whose experience could be used ....”

#### 3.3.2 Lack of attention to public education

According to most participants, despite previous flood experiences, there has never been public education for early warning, emergency evacuation and flood relief.

“More problems are related to the lack of awareness, if people are informed in advance, they will be less harmed in times of crisis ...”

### 3.4 Emergency housing problems

The problems related to emergency housing and shelter were also mentioned many times by the participants as a challenge.

#### 3.4.1 Lack of accommodation schedule

According to most of the participants, the lack of emergency accommodation camp, inadequate accommodation facilities, temporary accommodation, incompatibility of accommodation with local culture, lack of safe places in advance, and lack of recognition of emergency exits were important problems of emergency evacuation.

“Most of the flood victims were housed in tents, which were not a good place to live due to the strong winds. The tents were not enough for family members ...”

#### 3.4.2 Ignoring the indigenous culture in the settlement

According to most participants, people preferred to live in the homes of relatives and friends instead of using tents because of local, ethnic, and cultural issues. Participants expected to be housed in prefabricated houses with adequate facilities.

“People are settling more in family homes because of family ties and ethnic affiliation ... the government should set up a permanent camp ...”

### 3.5 Lack of risk perception

Many participants believed in the effect of lack of risk perception on evacuation.

#### 3.5.1 People believe in floods

According to most citizens, the depth and breadth of the risk was not understood because people had not experienced widespread flooding in the past. The previous mentality, as well as the previous false warnings, caused disbelief in the terrible and destructive flood.

“We had never seen such a flood in our lives ... we thought we could save our lives by going to the roof ....”

#### 3.5.2 Managers believe in floods

According to most managers, due to the lack of severe flooding in the past, the idea of extensive flood damage was not conceivable, so some departments did not evacuate. Observing this behavior by the people could not convince them to evacuate.

“.... Most of the offices were not evacuated and tried to control the flood by placing a few bags of sand next to the destroyed edges of the dam ... When people see this, they may not think it is a serious danger......”

### 3.6 Problems of incoordination

Problems related to incoordination were mentioned as challenge in flood evacuation. Participants pointed more to 234 times.

#### 3.6.1 Intra-organizational inconsistency

Most participants acknowledged that the lack of local crisis managers familiar with regional risks was one of the reasons for the lack of coordination within the organization.

“Our crisis managers are not aware of our hazards .... In most departments there is no plan to respond to the flood..... The organizations themselves are confused during the crisis .....”

#### 3.6.2 Extra-organizational inconsistencies

According to the participants, it was inferred that there was inconsistency in decisions in all organizations. The 2019 Poldokhtar flood coincided with the Nowruz holidays, and there was no written plan to coordinate departments to respond to possible holiday crises. Lack of pre-determined tasks, lack of anticipation of necessary equipment and delay in arrival of rapid response teams were some of the problems of emergency evacuation.

“Organizations involved in the crisis were either working in parallel or uncoordinated ...... We did not have a single command system ... The problem is in the crisis management structure ... We had the explosion of the polyethylene line and the fire at the same time as the flood....”

## 4 Discussion

This study aims to explore the challenges of evacuation in floods in Iran. Problems based on the views of citizens and executives include lack of coordination, lack of early warning system, insufficient resources, problems related to unpreparedness, problems related to emergency accommodation and lack of risk perception.

One of the challenges of this study was the lack of an early warning system. Liu et al. emphasized the importance of flood warning and information and considered the existence of early warning systems as a factor in reducing flood risk [26]. Fakhruddin’s study showed that 1-10-day forecasts and early warnings in Bangladesh may have a wide range of options for decision-makers, especially in the field of agriculture [27]. Parker showed that trust in flood warning methods affects how people prepare and respond to warnings. Lack of risk perception, lack of trust in officials and lack of participation in planning can be the reasons for the failure of flood warning programs. The role of government is very important in increasing public trust in news and media [28].

Insufficient resources were another challenge of evacuation. In other studies, the existence of financial resources for flood management has been considered [29, 30]. Timely and safe evacuation during floods requires advanced technology and necessary equipment [31].

In this study, unpreparedness was one of the problems of evacuation. Late flood notification increases mortality. Flood management and early warning are the responsibilities of governments, but public education can influence people’s behavior for emergency evacuation [30]. Emergency evacuation training before a flood occurs is effective in flood management. Crisis managers can increase flood preparedness by practicing, maneuvering, and simulating [32]. In this study, problems related to emergency accommodation were one of the problems of emergency evacuation. Studies show that adaptation to extreme weather events can be controlled with proper planning. Empowering vulnerable communities is possible through raising awareness, training rescue measures, providing infrastructure and strengthening social institutions to reduce disaster risk [33]. Chang also mentioned the lack of proper management of decision makers and the lack of forecasting of the required conditions during floods as important problems in flood management [34]. Twigg noted the improvement of the communication and information structure as well as the empowerment of crisis managers to make decisions in emergencies [35]. The existence of an operational plan during preparedness is an important point that can be useful for communities when responding to disasters [36]. Another way to increase disaster preparedness is to have periodical exercises [37, 38, 39, 40, 41, 42, 43, 44].

In this study, emergency housing problems was one of the problems of evacuation. One of the essential needs of people in the flood response phase is to prepare a suitable shelter for people, but despite this, it is not possible to provide adequate and suitable settlement [45].

In this study, the lack of risk perception in people and managers was one of the challenges of emergency evacuation. Awareness of risk is the first priority in the Global Disaster Reduction Program. If risk perception interventions are integrated at different levels of management, it can have a positive impact on community participation in disaster risk reduction. Increasing risk awareness reduces disaster damage and reinforces preventative behaviors [46].

In this study, problems related to lack of coordination of organizations were other challenges of emergency evacuation. Crisis management requires the cooperation of organizations and the existence of an incident command system [47]. Yari et al. Proposed comprehensive strategies to reduce flood risk. These include planning, training, raising awareness and understanding of risk, protecting vulnerable people, assessing flood risk, and improving flood management by responsible organizations [48].

### 4.1 Limitation and strength of the study

This study is the first qualitative study in the field of emergency evacuation experiences. The results of the data are collected from semi-structured interviews, so it is considered subjective. In future studies, it is recommended to use both quantitative and qualitative studies. The absence of female senior managers in crisis management and the coincidence of interview time with the Covid 19 epidemic is one of the limitations of the study.

## 5 Conclusion

Identifying challenges is essential to improving the emergency evacuation process. Emergency evacuation requires the coordination of responsive organizations. On the other hand, creating a national early warning system, providing human/communication/financial resources, conducting scenario-based exercises and increasing the level of citizens' awareness are effective in emergency evacuation.

## Declarations

### Author contribution statement

Changiz Ahmadi, Arezou Karampourian & Mohammad Reza Samarghandi: Conceived and designed the experiments; Performed the experiments; Analyzed and interpreted the data; Contributed reagents, materials, analysis tools or data; Wrote the paper.

### Funding statement

Changiz Ahmadi was supported by 10.13039/501100004697Hamadan University of Medical Sciences [IRUMSHA.REC.1400.039].

### Data availability statement

Data associated with this study has been deposited at “Hamadan University of Medical Sciences” under the accession number 0098-81-38380562.

### Declaration of interest’s statement

The authors declare no conflict of interest.

### Additional information

No additional information is available for this paper.
